# Fabrication and Performance of Aluminum-Based Composite Wicks Using a Two-Step Laser-Sintering Process

**DOI:** 10.3390/mi16040370

**Published:** 2025-03-25

**Authors:** Yong Tang, Yuxin Wei, Tong Sun, Jingjing Bai, Fangqiong Luo, Huarong Qiu, Yiming Li, Wei Yuan, Shiwei Zhang

**Affiliations:** 1 School of Mechanical and Automotive Engineering, South China University of Technology, Guangzhou 510640, China; ytang@scut.edu.cn (Y.T.); 202220100184@mail.scut.edu.cn (Y.W.); mentsun@mail.scut.edu.cn (T.S.); me_chesbai@mail.scut.edu.cn (J.B.); 202310180058@mail.scut.edu.cn (F.L.); mehrqiu@mail.scut.edu.cn (H.Q.); 19924686672@163.com (Y.L.); mewyuan@scut.edu.cn (W.Y.); 2 SCUT-Zhuhai Institute of Modern Industrial Innovation, Zhuhai 519175, China

**Keywords:** laser processing, wick structure, microgroove, capillary performance, spiral woven mesh

## Abstract

The evolution of 5G technology necessitates effective thermal management strategies for compact, high-power devices. The potential of aluminum-based vapor chambers (VCs) as thermal management solutions is recognized, yet the heat transfer performance is limited by the capillary constraints of the wick structures. This study proposes a laser-sintered composite wick to address this limitation. Experimental evaluations were conducted on microgroove wicks (MW) and groove–spiral woven mesh composite wicks (GSCW), utilizing ethanol and acetone as the working fluids. The MW, characterized by a laser spacing of 0.2 mm and two passes, demonstrated a capillary rise of 52.90 mm, while the spiral woven mesh (SWM) achieved a rise of 61.48 mm. Notably, the GSCW surpassed both configurations, reaching a capillary height of 84.57 mm and a capillary parameter ($K/R_{eff}$) of 2.769 μm, which corresponds to increases of 90.15% and 43.76% over the MW and SWM, respectively. This study demonstrates an effective approach to enhancing the capillary performance of aluminum wicks, which provides valuable insights for the design of composite wicks, particularly for applications in ultra-thin aluminum VC.

## 1. Introduction

As electronic components develop towards miniaturization, high integration, and increased power, the heat generated during their operation has escalated significantly. Limited heat dissipation space and increasing heat flux intensify the thermal challenge, underscoring the urgent need for effective thermal management in electronic devices [1,2]. Conventional air and liquid cooling techniques exhibit limitations in managing the thermal demands of high heat flux density applications, thus impacting the operational stability and longevity of electronic systems [3,4,5]. Vapor chambers, functioning as heat transfer devices that utilize phase change, are extensively employed in fields including electronic devices [6,7], aerospace [8,9], and fuel cells [10,11]. Within these systems, the role of the wick is pivotal for phase-change enhancement, as it facilitates working-fluid circulation via capillary action within the vapor chambers [12]. Wicks with superior capillary performance ensure the flow of the working fluid from the condenser to the evaporator within the vapor chamber. Micro/nanostructured wicks, known for their exceptional capillary performance, are especially important in vapor chambers [13,14]. Despite extensive research into the thermal conductivity and other heat transfer characteristics of vapor chambers, there is still a relative lack of in-depth analysis of the capillary behavior of wicks [15,16]. For wicks with fixed dimensions, the capillary performance parameters $K/R_{eff}$ are crucial in determining the capillary rise limit. Balancing the permeability K and pore diameter $R_{eff}$ of the wick to achieve optimal capillary performance presents a significant challenge and constitutes a central focus of this study.

Researchers have employed various processing and surface treatment techniques to manufacture high-performance wicks, including additive manufacturing [17,18], laser processing [19,20], and superhydrophilic treatments [21,22]. Chang et al. [21] employed a low-cost microsecond pulse laser micromachining method for the fabrication of super hydrophilic–super hydrophobic grooves as receptor sites for capillary self-alignment of microfibers, which modified the wetting properties of the microgrooves from 10° to 120° in terms of the contact angle. Liu et al. [23] controlled the etched substrate topography to modulate the morphology and capillary dynamics of the electrodeposited porous films on a specifically treated Cu substrate. After an optimal SPS etching treatment, the electrodeposited Cu film achieved a $K/R_{eff}$ value of 0.975 μm. Huang et al. [24] investigated the impact of different sintering temperatures on the capillary rise height of spiral mesh wicks. After 150 min of thermal oxidation, the wicks sintered at 400 °C showed a 32.2% increase in capillary rise height compared to the non-oxidized wicks. Following a one-hour reduction treatment, the capillary rise height reached 110% of that of a single sintered wick. Although copper-based wicks have been extensively studied for wick design and fabrication, the high cost and greater weight of copper restrict further development and application relative to alternative materials. Given the demand for efficient heat dissipation in confined spaces, lightweight and high-efficiency phase-change heat transfer devices have emerged as a focal point in current research.

Aluminum, due to its cost-effectiveness, high production scalability, and ease of industrialization, has become the preferred material for vapor chambers in various electronic applications. He et al. [25] utilized a one-piece extrusion process to manufacture an ultra-thin aluminum vapor chamber equipped with supporting pillars and microgrooves. The serrated microgrooves enhanced the heat transfer performance of the vapor chamber, achieving a minimum thermal resistance of 0.508 °C/W at a 30° inclination angle. Lou et al. [26] fabricated a nano-capillary aluminum finned heat sink, including surface modification and system design. For the first time, that allowed commercially available aluminum finned heat sinks to perform capillary-driven water evaporation from entire surface of fins for significant cooling enhancement. Aluminum flat-plate vapor chambers with microgrooves achieve high heat transfer rates and isothermal performance through interconnected gas–liquid channels [27]. These lightweight and simply structured aluminum flat-plate vapor chambers can be directly integrated with electronic components, providing an effective solution to thermal management challenges [28]. One of the primary limitations to enhancing vapor chamber performance is the restriction imposed by capillary flow and phase-change instability within the wick structure. Aluminum powder-sintered wicks present challenges for large-scale applications within vapor chambers due to issues such as the formation of aluminum oxide films and permeability constraints [25]. Groove-type wick structures are simpler, exhibit higher permeability, and are easier to manufacture. However, their lower capillary pressure can impact overall heat transfer performance within a vapor chamber. Composite wicks can significantly enhance capillary forces. However, research into aluminum composite wicks remains limited.

Nanosecond pulsed-laser machining, a rapid technique for improving surface topography, is widely used in fabricating wicks for vapor chambers. This method is particularly effective in creating microstructures on aluminum that exhibit hydrophilic capillary characteristics. He et al. [29] employed a two-step processing method, initially rolling to form Ω-shaped grooves, followed by laser scanning to create microgrooves, resulting in an aluminum wick with hierarchical grooves. This process significantly enhanced the capillary effect on the aluminum surface, achieving a maximum capillary rise height of 88.5 mm. Jiang et al. [30] utilized a two-step laser-machining process to fabricate a wick with dual-scale microgrooves. The capillary performance parameter (*K*/*R*_*eff*_) of the dual-scale microgrooves improved by 11.3% compared to single-scale microgrooves, reaching 1.322 μm. Chen et al. [31] fabricated superhydrophilic (SHPi) and superhydrophobic (SHPo) background surfaces on aluminum sheets using a nanosecond fiber laser. The capillary performance parameters reached 4.62 × 10^−7^ N, which was 117.9% higher than the pristine background surface. This study presents fresh suggestions for increasing the capillary performance of vertically grooved wicks. Liang et al. [32] fabricated a 500 mm long aluminum flat vapor chamber, incorporating a spiral woven mesh within the grooved channels to assist in the flow of the working fluid. This design achieved a low thermal resistance of 0.25 °C/W and an excellent thermal conductivity of 13,880 W/mK. In the aforementioned studies, the structures of aluminum-based vapor chambers were predominantly simple extruded microgroove designs, with limited research focused on the internal wick structures. Research on aluminum-based wicks remains limited, particularly in terms of understanding the impact of various composite structures on capillary performance and the absence of comparative testing with different working fluids. These gaps have created challenges in characterizing capillary phenomena and applying complex morphologies in composite porous structures. This study addresses these deficiencies by comparing the structures and morphologies of composite wicks and using ethanol and acetone as working fluids to gain deeper insights into the capillary performance of different composite wick structures.

This study employed a two-step laser-sintering process to fabricate aluminum-based microgroove wicks (MW) with varying laser parameters through a low-cost and straightforward method. Additionally, a groove–spiral woven mesh composite wick (GSCW) was fabricated by sintering a spiral woven mesh (SWM) into the grooves. Capillary rise experiments were conducted on all samples using ethanol and acetone as the working fluids to examine the effects of the laser parameters (scan-line spacing and scanning times) on the capillary performance of MW. The GSCW in this study successfully integrated different porous structures, reducing the resistance to capillary rise while providing additional storage channels. The capillary rise height reached 84.57 mm in ethanol and 70.20 mm in acetone. To more intuitively characterize the wicking capacity of GSCW, its highest capillary performance parameter (*K*/*R*_*eff*_) in the ethanol experiment reached 2.769 μm, representing 90.15% and 43.76% improvements over MW and SWM without composite structures and achieving an outstanding level in the current research on aluminum wicks. This study explored the mechanism of composite porosity and highlighted the promising potential of aluminum-based composite wick structures in enhancing the performance of aluminum-based vapor chambers. Future work could refine these structures to optimize their efficiency in high-performance cooling applications.

## 2. Manufacture and Experiments

### 2.1. Manufacture of Samples

**Figure 1:**
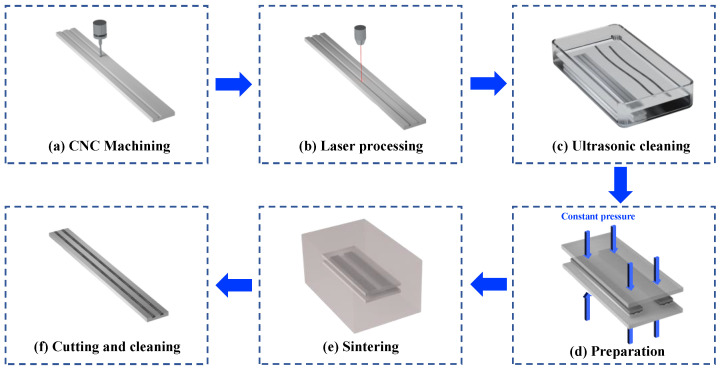
Manufacturing process of MW and GSCW samples. (**a**) CNC machining. (**b**) Laser processing. (**c**) Ultrasonic cleaning. (**d**) Preparation. (**e**) Sintering. (**f**) Cutting and cleaning.

In this study, 6063 aluminum alloy was selected as the substrate material for the MW samples due to its low cost, light weight, and superior mechanical properties. Concurrently, a spiral woven mesh made from 5154 aluminum alloy with a mesh density of 100 was chosen as the wick material for the SWM. The fabrication process of the samples is shown in Figure 1. Using computerized numerical control (CNC) machining techniques, two rectangular grooves with a width of 2.5 mm and a depth of 1 mm were created on the sample substrate, as illustrated in Figure 1a. The 5154 aluminum wire is subjected to the spiral-weaving process, where it is interwoven according to the predetermined spiral structure. The weaving process involves alternating the wires at specific angles and densities to form the spiral structure. The spiral woven mesh can then be trimmed and cut as needed to adjust the size and shape of the experiment samples. Subsequently, the MW and GSCW were fabricated through a two-step process involving nanosecond pulse laser machining followed by solid-state sintering.

In the initial phase, a V-shaped microgroove was created on the sample substrate through laser machining to produce the MW samples. Nanosecond pulsed-laser machining is an efficient technique for enhancing surface topology and is extensively employed in fabricating wicks for vapor chambers. This method effectively generates hydrophilic microstructures on aluminum, optimizing capillary performance. The nanosecond pulse laser system employed in this experiment operated at a power of 30 W and a wavelength of 1064 nm. The laser-machining parameters selected for this study included scanning line spacings of 0.1 mm, 0.2 mm, and 0.3 mm, with one and two scanning passes, and a laser-scanning speed of 50 mm/s. During the laser processing of microgrooves, an excessively fast scanning speed (e.g., 100 mm/s) may result in insufficient processing of the MW samples, preventing the complete formation of the longitudinal microgrooves. Conversely, an excessively slow scanning speed (e.g., 20 mm/s) fails to generate a rough and porous groove structure. Based on laser-processing experience, a scanning speed of 50 mm/s was selected as the optimal and standardized parameter for all MW samples. The MW samples were fabricated by laser processing within 2.5 mm wide rectangular grooves. To differentiate between the MW samples with varying laser-processing parameters, the samples with laser-scanning distances of 0.1 mm, 0.2 mm, and 0.3 mm, each subjected to two scanning passes, were designated as MW-12, MW-22, and MW-32, respectively. Additionally, the sample with a laser-scanning distance of 0.2 mm and a single laser-scanning pass was labeled as MW-21. This naming scheme was used to distinguish between MW samples with different laser-processing parameters. The laser processing of the MW samples is depicted in Figure 1b. Subsequently, the SWM was cut into strips measuring 2.5 mm in width and 110 mm in length. Both the MW and SWM samples underwent 10 min of ultrasonic cleaning using an aluminum surface oxide cleaner, ethanol, and deionized water sequentially in an ultrasonic cleaner (JP-020 Skymen Co., Shenzhen, China), as illustrated in Figure 1c.

In the second step, the laser-machined MW samples were assembled with the cleaned SWM and compressed using a graphite mold, as illustrated in Figure 1d. A high vacuum was achieved in the sintering chamber using mechanical and molecular pumps. Subsequently, the SWM and MW were subjected to solid-state sintering at 585 °C for 2 h under pressure applied by the mold, as shown in Figure 1e. The GSCW samples were fabricated by combining the MW samples with microgrooves and the SWM samples with a spiral woven structure. The two distinct porous structures were integrated through a sintering process. During the sintering process, a certain pressure was maintained on the samples using the graphite mold and stainless-steel fixtures. After sintering, the GSCW samples were separated from the graphite mold and trimmed to match the length of the aluminum alloy substrate, as illustrated in Figure 1f. Before the capillary rise test, the MW, SWM, and GSCW samples were ultrasonically cleaned using ethanol and deionized water. Subsequently, all samples were dried in a constant-temperature drying oven set at 50 °C to remove any residual moisture.

### 2.2. Characterization and Measurements

The surface morphology of the microgrooves on the MW samples post-laser processing was captured and analyzed using a three-dimensional profilometer (RTEC UP Dual Model, Nanjing, China). Additionally, the micro/nanoporous structures of the MW and GSCW samples were characterized using scanning electron microscopy (SEM, SU8600 Hitachi High-Tech (Shanghai) Co., Ltd. Shanghai, China). In the capillary rise experiment, the working fluid was driven by capillary pressure. Due to the complex surface morphology and structural variations among different wicks, accurately observing the precise position of the working fluid during the capillary rise process was challenging. Therefore, an infrared thermal imager (FLIR A615, FOV 15°, Shenzhen, China) and a fluorescence calibration method were employed in this study to characterize the liquid-level height during the capillary rise test. According to the Stefan–Boltzmann law, the infrared radiation energy of an object is related to its surface emissivity and temperature [26,33]. The infrared thermal imager captured the surface of the object to obtain data on the infrared radiation energy distribution, which was subsequently rendered into thermal images using computer software. By comparing the liquid surface heights at different times, the capillary rise curves for various samples were obtained. These curves were subsequently used to fit, calculate, and compare the relevant capillary performance parameters. The surface structures of the MW, SWM, and GSCW samples were complex, leading to differences in emissivity, which resulted in a less distinct liquid level contrast in the thermal images. Therefore, a fluorescence calibration method was employed for the capillary rise test. The samples in the fluorescence test were used only once, specifically to obtain accurate capillary rise height data. By adding a fluorescent dye to the working fluid and irradiating the samples with ultraviolet light, the capillary rise height was precisely measured. To ensure the reliability of the experiment, different samples were fabricated using the same process and tested under identical conditions.

**Figure 2:**
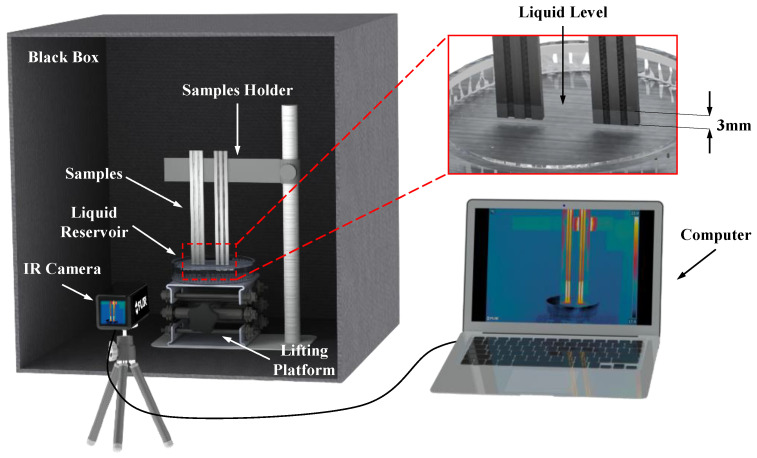
Schematic diagram of the capillary rise test setup.

As illustrated in Figure 2, the capillary rise test setup comprised three main components: a test bench, an infrared thermal imager, and a computer. The test device comprised a single-sided open black box, a lifting platform for sample placement, a stage for controlling the movement of the working fluid, and a glass dish. In the testing section, the sample was secured on a vertically adjustable lifting platform to control its initial height. A glass dish was used to contain the liquid, and the lifting platform was employed to adjust the vertical movement of the working fluid to ensure contact with the test samples. For the imaging section, an infrared thermal imager (FLIR A615, FOV 15°, Shenzhen, China) and an optical camera were used to capture the capillary rise process at a 4 K resolution and 60 fps frame rate. These devices were connected to a computer to obtain and analyze the captured images, facilitating detailed observation of the capillary rise.

In the working-fluid section, deionized water tended to react with aluminum due to its poor compatibility, rendering it unsuitable as the working fluid in aluminum vapor chambers. Ethanol and acetone were selected as the working fluids, with ethanol chosen for its high latent heat of vaporization and acetone for its low liquid viscosity. These characteristics make both fluids particularly well-suited for applications in phase-change heat transfer devices. Given that both ethanol and acetone are highly volatile, the glass dish containing the working fluid was positioned in the black box test environment 30 min before the capillary rise experiment. This step allowed the working fluid to evaporate and saturate the test environment, thereby reducing or eliminating the impact of fluid evaporation during the experiment. Additionally, during the capillary rise test of the wick samples, the small amount of working fluid stored in the wicks rendered the evaporation effect negligible.

During the testing, all samples were vertically fixed on the sample holder, ensuring adequate spacing between the samples and that the bottom ends of all samples remained at the same horizontal level. A ruler was positioned vertically beside the samples for calibration during data processing. Subsequently, by adjusting the elevation of the working-fluid platform, the bottom ends of the samples were simultaneously immersed 3 mm below the surface of the working fluid and then held stationary. When the working fluid made contact with the wick, the liquid spontaneously and rapidly rose due to capillary pressure, continuing until the liquid level remained unchanged for an extended period. At this point, it was considered that the capillary rise process was complete, and the working fluid had reached the maximum capillary rise height for the wick. Throughout this process, an infrared thermal imager or optical camera was used to record the event in real time, ensuring the accurate documentation of the capillary rise height.

### 2.3. Theory and Data Reduction

The wicking capability of composite wicks plays a critical role in the thermal performance of aluminum-based vapor chambers. For wire-mesh wicks and three-dimensional spiral woven-mesh wicks, directly measuring their permeability and capillary pressure presents significant challenges. The pore size, wire diameter, and mesh count of these wicks vary depending on the dimensional parameters, and factors such as clamping force and sintering temperature during sample preparation also differ. Given the unique characteristics of each wick type, it is challenging to draw direct comparisons or conclusions regarding their performance without considering these variables. These factors all exert varying degrees of impact on the wicking capability of the wicks. Therefore, this experiment conducted capillary rise tests on MW, SWM, and GSCW samples and evaluated their wicking capability using the capillary performance parameter $K/R_{eff}$. The capillary performance parameter $K/R_{eff}$ has been widely used to assess the wicking capability of different wicks in related studies [34,35]. The derivations of Equations (8) to (10) will be presented in detail in the following sections [36,37].

During the capillary rise process, based on the momentum balance, the pressure drop ($\Delta P_{drop}$) is equal to the sum of the viscous frictional pressure loss ($\Delta P_{f}$) and the hydrostatic pressure loss ($\Delta P_{g}$). The pressure balance in the capillary-wicking process could be expressed as follows:

(1) $$\Delta P_{drop}=\Delta P_{f}+\Delta P_{g}$$

According to the Young–Laplace capillary equation [38], the capillary pressure could be calculated by the following equation:

(2) $$\Delta P_{cap}=\frac{2\sigma }{R_{eff}}$$

where σ is the surface tension of the liquid. $R_{eff}$ is the effective pore radius, which could be expressed in terms of the pore radius $R_{p}$. The contact angle θ is as follows:

(3) $$R_{eff}=\frac{R_{p}}{cos\theta }$$

According to Darcy’s law [39], the viscous frictional pressure drop could be calculated by the following equation:

(4) $$\Delta P_{f}=\frac{\mu \varepsilon }{K}h\frac{dh}{dt}$$

where μ is the dynamic viscosity of the fluid, K is the permeability of the wicks, h is the capillary rise height, $\frac{dh}{dt}$ is the wicking rise rate, and ε is the porosity of the wick structure, which could be calculated by the following equation:

(5) $$\varepsilon =\left(1-\frac{V_{twa}}{V_{tw}}\right)\times 100\%$$

In the equation, $V_{twa}$ represents the total volume of all of the aluminum woven tapes in the wicks. The calculation formula is as follows:

(6) $$V_{twa}=n_{swm}V_{as}=n_{swm}\frac{\pi }{4}{d_{as}}^{2}L_{as}n_{as}$$

The permeability of the wicks could be calculated or predicted using the Blake–Kozeny equation [40], given by:

(7) $$K=\frac{{d_{p}}^{2}\varepsilon^{3}}{122{\left(1-\varepsilon \right)}^{2}}$$

The hydrostatic pressure loss could be calculated by the following equation:

(8) $$\Delta P_{g}=\rho gh$$

where *ρ* is the density of the liquid, *g* is the acceleration due to gravity, and *h* is the capillary rise height of the liquid. When the capillary pressure, viscous frictional pressure, and hydrostatic pressure reach equilibrium, the capillary rise process ceases [41]. During the capillary rise, inertial effects could be neglected, and the capillary pressure equals the pressure drop. Therefore, the equation could be rewritten as:

(9) $$\Delta P_{drop}=\Delta P_{cap}$$

By integrating Equations (2), (4), (8), and (9), Equation (1) could be rewritten as follows:

(10) $$\frac{2\sigma }{R_{eff}}=\frac{\mu \varepsilon }{K}h\frac{dh}{dt}+\rho gh$$

In the initial stage, the effect of gravity could be neglected [42]. Therefore, the equation could be solved under the initial condition h(t→0)=0. The result is:

(11) $$h^{2}=\frac{4\sigma }{\mu \varepsilon }\frac{K}{R_{eff}}t$$

(12) $$h=\sqrt{\frac{4\sigma K}{\mu \varepsilon R_{eff}}\times t}=W\sqrt{t}$$

It is evident that, during the initial phase, the h is linearly related to the square root of time, where the W is the wicking coefficient, an important parameter for measuring wicking capability, and also represents the liquid propagation capability [43].

### 2.4. Uncertainty Analysis

The measurement uncertainties for the width w, thickness δ, and capillary rise height h of the aluminum spiral woven mesh are within 2.0%, 1.0%, and 2.0%, respectively. Using the standard error analysis method, the measurement uncertainties of the porosity ε, wicking coefficient W, and the capillary performance parameter $K/R_{eff}$ were calculated (Table 1).

## 3. Result and Discussion

### 3.1. Surface Morphology Characterization

Porosity and capillary rise height are crucial parameters for characterizing the capillary performance of wick structures. In this study, the microporous structure of MW and GSCW samples directly influences their capillary performance. Therefore, the focus was on investigating the laser-processing parameters of MW samples and the composite pore structure of GSCW samples. Extensive research has demonstrated that the fabrication of surface microstructures on metal surfaces through laser processing could significantly enhance capillary performance. During the laser processing, an ultra-high energy laser beam is applied to the surface of the rectangular grooves on the aluminum substrate, causing the surface to melt. As the laser focal point moves, multiple longitudinal parallel microgrooves are generated on the sample surface. The interaction between aluminum and oxygen during laser processing results in the creation of textured microgrooves. These laser-induced microgrooves instantly exhibit superhydrophilic properties [44].

**Figure 3:**
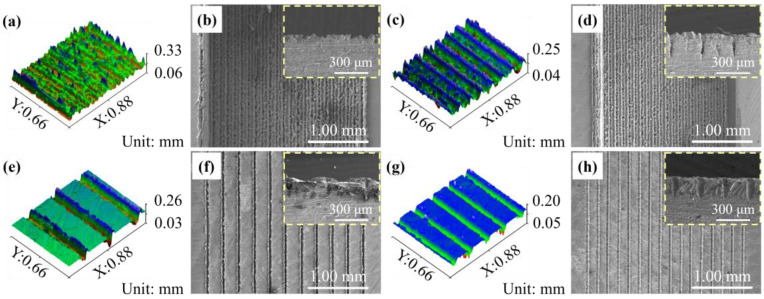
3D profilometry and SEM top and cross-sectional images of MW samples with different laser line spacings: (**a**,**b**) MW-12 sample. (**c**,**d**) MW-22 sample. (**e**,**f**) MW-32 sample. (**g**,**h**) MW-21 sample.

Figure 3a–h illustrate the 3D surface profiles and corresponding SEM front and cross-sectional views of the four MW samples processed with varying laser scan-line spacings. From the 3D profiles and SEM images, it is evident that laser processing produces V-shaped grooves approximately 40 μm wide and 160 μm deep within the rectangular grooves of the samples. As shown in Figure 3a,c,e,g, when the laser scan-line spacing is 0.1 mm, and the closely spaced ridges of adjacent microgrooves on the MW-21 sample surface interlock and merge, failing to form distinct, parallel grooves with uniform height on a single plane, leading to an irregular pattern. As the laser scan-line spacing increases, the microgrooves formed with 0.2 mm and 0.3 mm spacings become more distinct and regular. Comparing samples MW-22 and MW-32, the larger scan-line spacing in MW-32 results in the absence of interaction between adjacent grooves, resulting in a noticeable gap between each parallel microgroove formed by the laser. When comparing samples MW-22 and MW-21, the difference in laser-processing times results in MW-21 lacking the compressive ridges formed by the high temperature of the laser at the edges of the microgrooves, leaving a gap between adjacent grooves.

As shown in Figure 3b, the SEM image of sample MW-21 exhibits splashing of molten alumina, which accumulates on the surfaces of micro-cavities. Therefore, the MW-21 sample’s surface is uneven and lacks uniform parallel grooves in the vertical direction. Figure 3d illustrates that the MW-22 sample, benefiting from an appropriate laser line spacing, exhibits clear, parallel adjacent microgrooves, with spacing between the raised molten materials formed by adjacent microgrooves approximately equal to the width of a single microgroove. This results in uniformly spaced grooves filling the entire rectangular groove surface. In Figure 3f, the MW-32 sample, processed with a laser scan-line spacing of 0.3 mm, shows clear and parallel microgrooves. However, the excessive spacing between the adjacent grooves precludes interaction or mutual influence, resulting in independent groove structures. Figure 3h shows that the MW-21 sample, with only a laser scan one time, forms shallow grooves on the surface. The molten alumina does not form pronounced ridges as in MW-22, leaving gaps between the adjacent grooves and resulting in shallow U-shaped grooves. Increasing the depth of V-shaped grooves and reducing the corner dimensions decreases the meniscus radius, thereby enhancing capillary pressure and capillary performance [45]. Therefore, the deeper V-shaped grooves of MW-12 and MW-22 endow these materials with more effective capillary structures. Conversely, the structure of MW-32 and MW-21 fail to offer significant improvements in capillary-driven fluid transport or liquid storage capacity.

**Figure 4:**
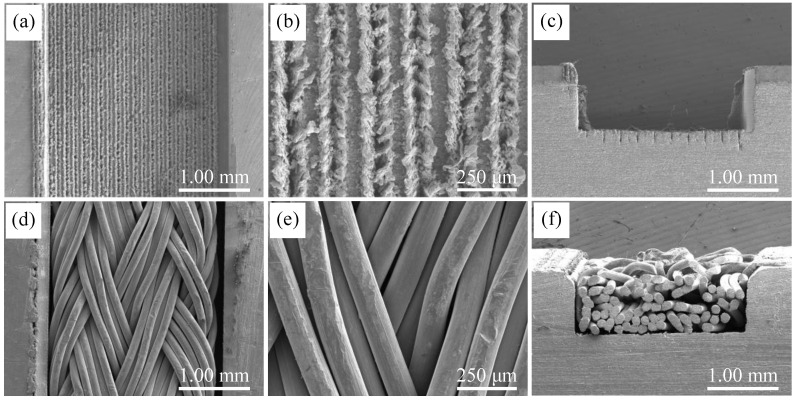
SEM images of (**a**–**c**) the MW sample. (**d**–**f**) The GSCW sample.

The SEM images of the MW and GSCW samples are compared in Figure 4. The surface structural characteristics of the GSCW sample are shown in Figure 4d, while the cross-sectional structural features are illustrated in Figure 4f. The aluminum SWM used in this study was fabricated from 5154 aluminum alloy, with each wire having a diameter of 0.11 mm. The braiding technique involved intertwining five wires to form a single strand, which was then helically braided. This SWM exhibited a complex porous structure, with pores formed by the intertwining of wires and by the compression of different strands. After sintering, the laser-processed grooves and surface protrusions became connected with the protrusions and SWM on their surfaces due to solid-phase sintering. The intertwining pores and the edges of the microgrooves on the surface of the SWM form new pores of varying sizes, enhancing channels for liquid storage and capillary action. This structural enhancement significantly improves the capillary performance of the GSCW.

### 3.2. Wicking Height Characterization

#### 3.2.1. Capillary Performance of Different Laser Parameters

**Figure 5:**
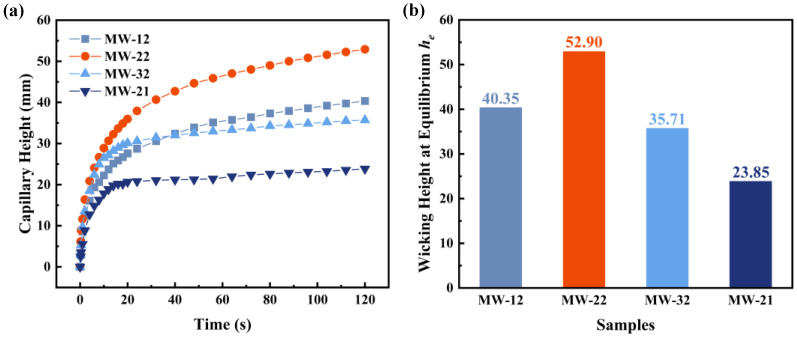
(**a**) Capillary rise curves of MWs tested with ethanol. (**b**) Wicking heights of MWs with varying line spacings at equilibrium.

A lightweight wick with superior capillary performance is crucial for the fabrication of high-performance heat pipes and vapor chambers, with the capillary rise height serving as a critical indicator of the anti-gravity performance of heat pipes [19]. To characterize the capillary performance of different MW samples, this study compares the capillary rise curves and final stabilized heights across various samples. Figure 5 illustrates the capillary rise curves and stabilized heights of four MW samples with varying laser parameters, with ethanol as the working fluid. As shown in Figure 5a, the capillary rise height of each wick rapidly increases during the initial stage, with the working fluid quickly ascending through the microscale grooves, which is consistent with theoretical predictions where gravitational influence on capillary pressure is negligible in the early phase. After 20 s, the capillary rise rates of all samples decrease as the wicking rate gradually levels off due to increasing viscous friction and gravitational effects [39], resulting in noticeable differences in capillary rise height. The wicking height reaches its maximum and stabilizes when the total pressure drop equals the capillary pressure, signifying the completion of the capillary rise process. Figure 5b illustrates the stabilized capillary rise heights of the four MW samples. The MW-12, MW-22, and MW-32 samples, subjected to two laser passes, exhibit capillary rise heights of 40.35 mm, 52.90 mm, and 35.71 mm, respectively. For the samples with a laser line spacing of 0.2 mm, the capillary rise heights for MW-21 and MW-22 are 23.85 mm and 52.90 mm, respectively. The MW-22 sample exhibits the highest capillary rise height, which is observed to be 31.10%, 48.14%, and 121.80% greater than those of the MW-12, MW-32, and MW-21 samples, respectively. These results indicate that the resultant porous microgrooves provide additional channels for the liquid capillary rise, demonstrating optimal capillary performance when the laser line spacing is 0.2 mm and the number of laser passes is two. This observation aligns with the characteristics observed in the 3D profilometry and SEM images, where adjacent microgrooves form novel porous structures.

**Figure 6:**
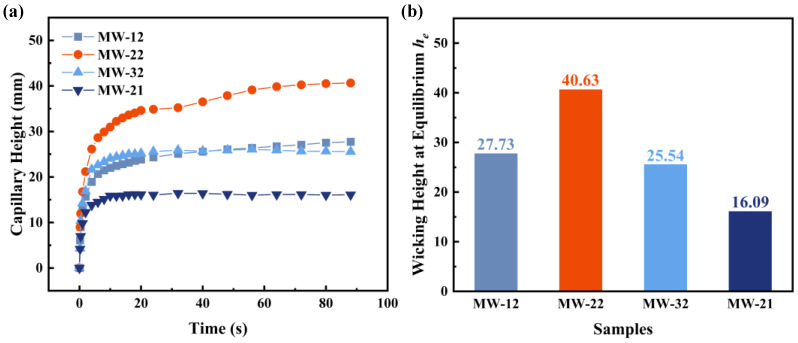
(**a**) Capillary rise curves of MWs tested with acetone. (**b**) Wicking heights of MWs with varying line spacings at equilibrium.

Figure 6 presents the capillary rise curves and final stabilized heights of the four MW samples using acetone as the working fluid. The results exhibit similar patterns and trends to those observed in Figure 5. When acetone is used as the working fluid, the initial rise rate is slightly higher than that observed with ethanol, although the final stabilized capillary rise height is lower. The capillary rise heights for MW-12, MW-22, MW-32, and MW-21 are 27.73 mm, 40.63 mm, 25.54 mm, and 16.09 mm, respectively. The capillary rise height of the MW-22 sample is 46.52% and 59.08% higher than those of MW-12 and MW-32, respectively, with the same number of laser passes, and 152.52% higher than that of MW-21 with the same laser line spacing. These results indicate that the MW-22 sample continues to exhibit the best capillary performance, which is consistent with the structural characteristics observed in the characterization.

Tests on the MW samples reveal that, with ethanol and acetone as the working fluids, the MW-22 sample shows the highest capillary rise rate and the greatest capillary rise height. This indicates that the microgroove structure formed under these laser parameters possesses the optimal porous structure for capillary performance, making it the most suitable microgroove structure for GSCW.

#### 3.2.2. Capillary Performance of Different Wicks

**Figure 7:**
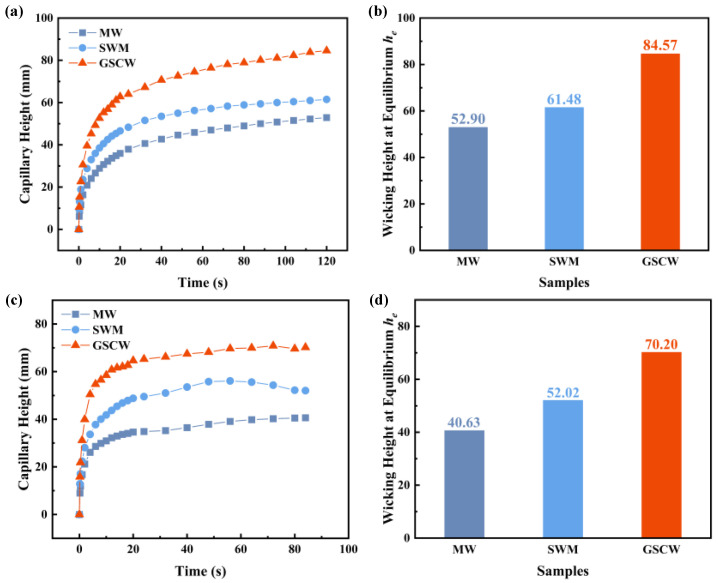
Capillary rise height and equilibrium wicking height of MW, SWM, and GSCW samples tested with ethanol and acetone. (**a**) Capillary rise with ethanol. (**b**) Equilibrium wicking height with ethanol. (**c**) Capillary rise with acetone. (**d**) Equilibrium wicking height with acetone.

Figure 7 illustrates the capillary rise results for three wick samples using ethanol and acetone as working fluids. As shown in Figure 7a,b, within the first 5 s of the ethanol test, GSCW rapidly ascends to a height of 40 mm. After 20 s, the capillary rise rates for all three samples decrease. The final capillary rise heights for the MW, SWM, and GSCW samples are 52.90 mm, 61.48 mm, and 84.57 mm, respectively. The complex and non-uniform V-shaped grooves in the MW sample create irregular voids and discontinuous channels, leading to additional horizontal capillary resistance as the liquid ascends. In contrast, the SWM sample features smaller, continuous pore channels formed between the aluminum alloy wires and larger channels between the different strands, allowing the liquid to rise continuously with reduced resistance. The SWM sample exhibits lower flow resistance and higher capillary force, resulting in a faster capillary rise rate. The GSCW sample demonstrates the fastest initial capillary rise rate and an ultimate rise height that is 59.87% and 37.56% higher than those of MW and SWM, respectively. In the ethanol test, the GSCW exhibits superior capillary performance. Figure 7c,d show the capillary rise results using acetone, revealing trends similar to those observed with ethanol. The initial capillary rise rate with acetone is faster than that with ethanol, but the final rise height is slightly lower. The final capillary rise heights for MW, SWM, and GSCW were 40.63 mm, 52.02 mm, and 70.20 mm, respectively. The final capillary rise height of the GSCW sample was 72.78% and 34.95% higher than those of the MW and SWM, respectively. The results indicate that the GSCW continues to exhibit superior capillary performance in acetone tests, which is consistent with the conclusions drawn from the structural characterization in Figure 4 and the capillary rise results in Figure 7.

### 3.3. Wicking Capability

**Figure 8:**
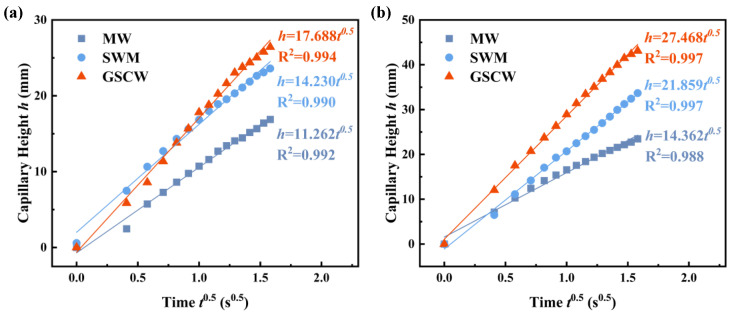
Wicking height h as a function of $t^{0.5}$ with (**a**) ethanol and (**b**) acetone.

To compare the capillary performance of the three samples, in addition to their respective final capillary heights, analyzing and comparing their capillary coefficient W and the capillary performance parameter *K*/*R*_*eff*_ could evaluate the comprehensive performance of the permeability *K* of wick structures and effective pore radius *R*_*eff*_. According to Equation (10), the initial 2 s capillary rise curves of the MW, SWM, and GSCW samples are fitted to a linear function of capillary height h versus $t^{0.5}$, with the slope representing the estimated value of the capillary parameter W, as shown in Figure 8. Based on the experimental data, the slopes for the MW, SWM, and GSCW samples in the ethanol tests are 11.262 $\mathrm{mm}/s^{0.5}$, 14.230 $\mathrm{mm}/s^{0.5}$, and 17.688 $\mathrm{mm}/s^{0.5}$, respectively. In the acetone tests, the slopes are 14.362 $\mathrm{mm}/s^{0.5}$, 21.859 $\mathrm{mm}/s^{0.5}$, and 27.468 $\mathrm{mm}/s^{0.5}$, respectively. In both testing fluids, the GSCW exhibits the highest fitted slope, indicating that its capillary coefficient W and capillary performance parameter *K*/*R*_*eff*_ are the highest among the three samples. This could be attributed to the optimal balance between capillary pressure and permeability and wettability of the grooves and SWM, demonstrating superior capillary performance among all samples.

### 3.4. Comparison with Previous Studies

**Figure 9:**
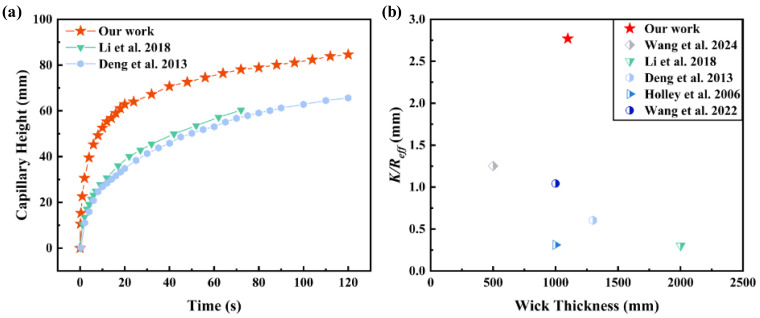
Comparison of (**a**) capillary height and (**b**) capillary performance parameters (*K*/*R*_*eff*_) of different wick structures with different thicknesses based on the studies by Wang et al. [46], Li et al. [47], Deng et al. [48], Holley et al. [49] and Wang et al. [50].

Compared to the existing research on wicks, an advanced wick design approach is presented in this study, which is designed by integrating MW with SWM, as illustrated in Figure 9. Inspired by the nutrient and water transport system of rice roots, Wang et al. [46] produced ultra-thin copper fiber wicks with an orderly arrangement through limited fiber sintering and hydraulic methods. After chemical treatment, the capillary performance parameter reached 1.254 μm. Li et al. [47] fabricated multi-scale composite porous wicks by constructing nanostructures on sintered copper powder using alloying–dealloying methods. The use of irregular copper powder significantly improved the capillary performance, achieving a parameter of 0.302 μm. Deng et al. [48] produced porous wicks by loose-sintering nickel and copper powders, with copper porous wicks, exhibiting permeability an order of magnitude higher than nickel and capillary performance parameters 1.5–2 times greater, reaching a maximum of 0.604 μm. Holley et al. [49] created wicks by sintering F-15 alloy powder at 1100 °C, resulting in samples with different particle size distributions and a capillary performance parameter of 0.311 μm. Wang et al. [50] used sintering and laser ablation to fabricate grooved wicks with multi-scale porous structures on silicon carbide materials, achieving high porosity and a capillary performance parameter of 1.042 μm. In summary, the GSCW in this study achieved high porosity and a composite effect using a relatively simple sintering process, reaching the highest capillary performance parameter *K*/*R*_*eff*_ of 2.769 μm. Compared to the microgroove structures and composite wicks in other studies, GSCW employed a simpler fabrication process and achieved a higher capillary performance parameter, making it more effectively applicable in an aluminum-based phase-change heat transfer device.

## 4. Conclusions

In this study, an innovative method for fabricating aluminum composite wicks was introduced. The MW and SWM were integrated by utilizing a laser-sintering two-step processing technique, resulting in composite wicks with novel porous structures. Capillary rise experiments were conducted using ethanol and acetone as working fluids on various samples. The GSCW achieved a maximum capillary rise height of 84.57 mm and a capillary performance parameter (*K*/*R*_*eff*_) of 2.769 μm. This study indicates that the GSCW demonstrates exceptional capillary performance, offering an effective solution for liquid circulation in ultra-thin aluminum-based vapor chambers. The specific conclusions are as follows:

(1) Laser processing of the aluminum alloy substrate enabled the precise fabrication of parallel porous microgrooves. The solid-state sintering technology effectively integrated the MW with SWM to form composite wicks with superior capillary performance;
(2) The MW-22 sample demonstrated the highest capillary performance among the MWs when the laser-scanning line spacing is 0.2 mm and the scanning passes are two. The capillary performance parameter of MW-22 reaches 1.456 μm, reflecting an increase of 58.21% compared to MW-21. This improvement is attributed to the formation of new microgrooves created by adjacent ridge-like protrusions in the MW, where parallel microscale grooves enhanced the capillary performance of wicks;
(3) The GSCW exhibited excellent capillary performance, with a maximum capillary rise height of 84.57 mm and *K*/*R*_*eff*_ of 2.769 μm when using ethanol as the working fluid. Compared to the non-composite MW, the capillary rise height and the capillary performance parameter *K*/*R*_*eff*_ were enhanced by 59.86% and 90.15%, respectively.

This study identified the optimal laser parameters for MW and developed a GSCW with exceptional capillary performance, providing valuable insights into designing wick structures with varying pore scales. However, further research is needed to design wick structures with more rational combinations and to explore the proper application in ultra-thin aluminum vapor chambers.

## Figures and Tables

**Table 1:** Uncertainty calculation results.

Calculated Parameter	Equation	Result
porosity ε	$\frac{e\left(f\right)}{f}=\frac{\sqrt{\sum {\left(\frac{\partial f}{\partial x_{i}}e_{x_{i}}\right)}^{2}}}{f}$	1.92%
wicking coefficient W		4.47%
$\mathrm{capillary}\;\mathrm{performance}\;\mathrm{parameter}\;K/R_{eff}$		4.28%

where e(f) represents the measurement uncertainty of the variable *f*, and $e_{x_{i}}$ is defined as the maximum measurement uncertainty for each component $x_{i}$. The uncertainties for porosity ε, wicking coefficient W, and $K/R_{eff}$ are 1.92%, 4.47%, and 4.28%, respectively.

## Data Availability

The original contributions presented in the study are included in the article, further inquiries can be directed to the corresponding author.

## Nomenclature

**Nomenclature**	
$d_{as}$	Diameter of each aluminum wire in the wick, m
$d_{p}$	Equivalent diameter of the wick, m
e(f)	Measurement error of the variable f.
g	Gravitational acceleration at the current state, m/s^2^
h	Height of the liquid working medium at the current state, m
$h_{e}$	Wicking height at equilibrium, m
K	Permeability of the wicking structure, m^2^
$L_{as}$	Length of the aluminum wire in the wick, m
$n_{as}$	Number of the aluminum wire in the wick
$n_{swm}$	Number of aluminum spiral woven mesh
$\Delta P_{drop}$	Pressure drop, Pa
$\Delta P_{cap}$	Capillary pressure, Pa
$\Delta P_{f}$	Viscous frictional pressure loss, Pa
$\Delta P_{g}$	Hydrostatic pressure loss, Pa
$R_{eff}$	Effective pore radius, m
$R_{p}$	Pore radius, m
t	Original measurement time, s
V	Volume, m^3^
$V_{as}$	Volume of the aluminum wire, m^3^
$V_{tw}$	Total volume of the wick, m^3^
$V_{twa}$	Total volume of all the aluminum spiral woven mesh, m^3^
W	Capillary coefficient, m/s^0.5^
**Greek symbols**	
ρ	Density of liquid working medium, kg/m^3^
σ	Surface tension of the liquid working medium, N/m
θ	Contact angle of the liquid on the wicking structure surface, °
ε	Porosity of the wicking structure
μ	Dynamic viscosity of the saturated liquid working medium, N·s/m^2^
**Abbreviations**	
MW	Microgroove wick
SWM	Spiral woven mesh
GSCW	Groove–spiral woven mesh composite wick
SEM	Scanning electron microscope
